# Benefits of camrelizumab plus carboplatin and albumin paclitaxel as induction therapy for locally advanced borderline resectable or unresectable esophageal squamous cell carcinoma

**DOI:** 10.1111/1759-7714.15232

**Published:** 2024-02-05

**Authors:** Li Chao, Jianting Liu, Yun Chen, Yuhui Fan, Shiping Guo, Shuangping Zhang

**Affiliations:** ^1^ Department of Thoracic Surgery, Shanxi Cancer Hospital Taiyuan PR China

**Keywords:** chemotherapy, immunotherapy, locally advanced esophageal carcinoma, neoadjuvant immune plus chemotherapy

## Abstract

**Background:**

To evaluate the safety and efficacy of camrelizumab plus albumin paclitaxel and carboplatin in the neoadjuvant treatment of borderline resectable or unresectable locally advanced esophageal cancer.

**Methods:**

A retrospective analysis was conducted on 27 patients with borderline resectable or unresectable locally advanced esophageal cancer who received neoadjuvant treatment with camrelizumab plus albumin paclitaxel and carboplatin at Shanxi Cancer Hospital from January 2020 to March 2022. Of these, 20 patients underwent thoracoscopic esophagectomy after neoadjuvant treatment.

**Results:**

Overall, 88.9% (24/27) of patients completed neoadjuvant treatment. The objective response rate was 79.2% (19/24) according to the RECIST criteria. Of the 20 patients who underwent surgery, the R0 resection rate was 95%, and 35% (7/20) achieved pathological complete response (pCR). During neoadjuvant treatment, 30% (6/20) of patients experienced grade ≥3 treatment‐related adverse events (TRAEs), and 20% (4/20) had grade ≥3 postoperative complications. There were no cases of reoperation or perioperative mortality.

**Conclusion:**

Camrelizumab plus albumin paclitaxel and carboplatin were found to be safe and effective in the neoadjuvant treatment of borderline resectable or unresectable locally advanced esophageal cancer. It was observed to improve the rate of curative resection without increasing perioperative complications.

## Graphical abstract

Of the 20 patients who received camrelizumab plus albumin paclitaxel and carboplatin as transformative therapy and underwent surgery, the R0 resection rate was 95%, and 35% (7/20) patients achieved pathological complete response (pCR).

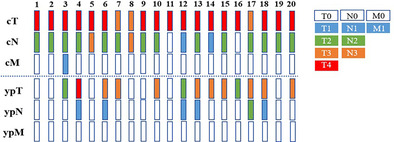

## INTRODUCTION

Esophageal cancer is a relatively common malignancy in China, accounting for more than half of the global incidence rate.^1^ Due to the lack of a mesentery layer in the outermost layer of the esophagus, tumors in the thoracic esophagus can easily penetrate the esophageal wall and invade surrounding organs, especially the trachea, bronchi, heart, and aorta. The Union for International Cancer Control (UICC) TNM classification defines tumors involving adjacent organs as T4, and these account for 12%–34% of thoracic esophageal cancer.^2^ For patients with locally advanced esophageal cancer considered unresectable at the initial diagnosis, the standard treatment is radical chemoradiotherapy (CRT). However, the complete clinical response rate is only 25%–32%, and 18%–22% of patients experience severe complications such as esophageal perforation and esophagotracheal fistula, which has caused widespread clinical concern.^3^ Since preoperative immunotherapy can stimulate the patient's immune system to recognize tumor antigens and form immune memory, and can also promote the continuation of immune surveillance after surgical removal of the tumor, current research is investigating the use of immunotherapy before surgery for various solid tumors.^4^, ^5^, ^6^ This has led to the exploration of new treatment strategies for perioperative esophageal cancer. Previous studies demonstrated that compared with single chemotherapy, improved efficacy was reported in esophageal cancer patients who received combination treatment with immunotherapy and chemotherapy.^7^ A phase II clinical trial of conversion therapy for unresectable locally advanced esophageal cancer conducted by Yokota et al. achieved an R0 resection rate of 39.6% with no postoperative complications or surgery‐related deaths.^8^

Camrelizumab is a humanized antiprogrammed cell death‐1 (PD‐1) antibody. Studies have shown that combinations of camrelizumab and chemotherapy could provide clinical benefits as first‐line treatment in patients with late‐stage or metastatic esophageal cancer, as well as neoadjuvant therapy in patients with locally advanced esophageal cancer.^9^, ^10^ However, the immunotherapeutic agents and combined chemotherapy regimens currently used vary, and there are relatively few related studies. The present study of camrelizumab in combination with albumin paclitaxel and carboplatin in borderline resectable or unresectable locally advanced esophageal cancer was a single‐arm, single‐center trial, in which we also demonstrated that locally advanced esophageal cancers that are not resected prior to surgery at R0 require preoperative transformative therapy, which is in line with previous studies^11^

Therefore, we conducted a single‐arm, single‐center retrospective analysis of 27 patients treated with camrelizumab combined with albumin paclitaxel and carboplatin preoperative chemotherapy and immunotherapy in our hospital, to evaluate the safety and feasibility of chemotherapy combined with immunotherapy using camrelizumab together with albumin paclitaxel and carboplatin in the real‐world setting of borderline resectable or unresectable locally advanced esophageal cancer.

## METHODS

### Patients

We included patients with locally advanced esophageal cancer who received treatment at Shanxi Provincial Cancer Hospital from January 2020 to October 2021. The inclusion criteria were: (1) Pathological confirmation of the diagnosis before surgery. (2) Preoperatively assessed as borderline resectable or unresectable locally advanced esophageal cancer (cT4 and/or cN3). (3) Patients who received camrelizumab combined with chemotherapy. Patients who were still receiving conversion therapy were excluded.

### Staging and evaluation

The segmentation and TNM staging of esophageal cancer were based on the eighth edition of the International Esophageal Cancer TNM Staging Standard, issued in 2017. Preoperative examinations included gastroscopy, pathological biopsy, chest and head enhanced CT scans, upper gastrointestinal contrast studies, bilateral neck, and abdominal color Doppler ultrasound, and, if necessary, endoscopic ultrasonography, esophageal magnetic resonance imaging (MRI), or positron emission tomography‐computed tomography (PET‐CT). For patients with large lesions or suspected tracheal invasion in the thoracic segment of the esophagus, fiber bronchoscopy was performed. After every two cycles of conversion therapy, chest CT scans and other examinations were conducted, and after 2–4 cycles of conversion therapy, the overall effectiveness of the conversion therapy was evaluated through comprehensive examinations according to the RECIST 1.1 criteria.

### Preoperative induction protocol

Camrelizumab: 200 mg per infusion, intravenously administered over 30 min (not less than 20 min and not more than 60 min). Albumin paclitaxel 260 mg/m^2^ and carboplatin area under the curve (AUC) = 3 were given on day 1 of each cycle. The treatment was administered every three weeks, and at least two cycles of preoperative treatment were required.

### Surgical approach

Surgery refers to the treatment of patients who had been evaluated as potential R0 resection candidates after disease conversion therapy. These patients underwent surgery after full consultation with the patients and their families. The surgical approach used was a thoracoabdominal laparoscopic esophageal cancer resection (McKeown) + two‐field lymph node dissection (including extended two‐ and three‐field lymph node dissection).

### Observation outcomes

The primary outcome was the pathological complete response rate (pCR). The secondary outcomes were the objective response rate (ORR), the R0 resection rate, safety, and perioperative complications. Adverse reactions to chemotherapy and immunotherapy were graded according to the NCI‐CTCAE4.0 grading criteria. The definition and classification of perioperative complications were based on the guidelines of the Society of Thoracic Surgeons and the General Thoracic Surgery Database. The Clavien complication grading system was used to grade complications. Clavien grades 1–2 were classified as mild complications, and Clavien grades 3–5 were classified as severe complications.

### Statistical analysis

SPSS 24.0 software (IBM Corporation) was used for statistical analysis. Baseline and safety analyses were performed for the enrolled patients, with continuous variables presented as mean ± standard deviation and categorical variables described using frequencies and percentages. A *p*‐value of <0.05 was considered statistically significant.

## RESULTS

### General features

From January 2020 to March 2022, a total of 27 patients were enrolled. Three patients refused further treatment after one cycle of therapy. A total of 22 patients completed two cycles of medication, one completed three cycles, and one completed four cycles. Two patients refused surgery after clinical remission, one patient was treated with apatinib, and one case is still awaiting conversion. A total of 20 patients underwent surgical treatment (Table 1).

**TABLE 1:** General information and patient clinicopathological characteristics.

Characteristics	No. (%)
Age, range	65.75 ± 6.03 (57–74)
Sex	
Male	8 (57.1%)
Female	6 (42.9%)
Tumor length, cm	6.52 ± 2.56 (3–14)
Tumor location	
Proximal third	1 (5%)
Middle third	17 (85%)
Distal third	2 (10%)
Clinical stage	
cIII	2 (10%)
cIVa	17 (85%)
cIVb	1 (5%)
ECOG	
0–1	14 (70%)
2	4 (20%)
3	2 (10%)
Chemotherapy + immunotherapy course	
2	18 (90%)
3	1 (5%)
4	1 (5%)

Abbreviation: ECOG, Eastern Cooperative Oncology Group.

### Surgical procedures and postoperative pathological evaluation

**FIGURE 1:**
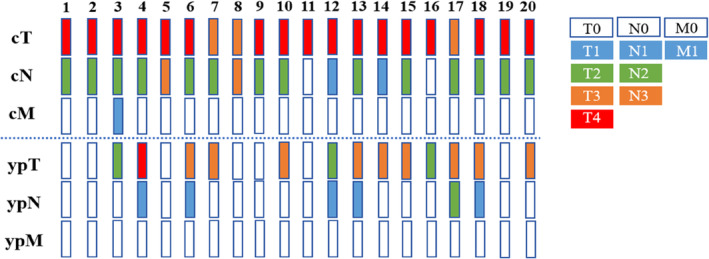
Preoperative clinical (cTNM) evaluation compared with the final pathological evaluation.

A total of 19 out of the 20 patients who underwent surgical treatment achieved R0 resection (95%), and one underwent palliative resection. A total of 18 patients underwent thoracoabdominal esophageal cancer resection and two‐field lymph node dissection, while one underwent thoracoabdominal esophageal cancer resection and three‐field lymph node dissection due to previous partial gastrectomy. This patient also underwent esophagogastrostomy with jejunal interposition in the left neck. None of the 20 patients required conversion to thoracotomy. The postoperative pathology showed that seven of the 20 surgical patients achieved complete response (5 cases of T0N0M0, 2 cases of TisN0M0), with a complete response rate of 35% (7/20). A total of 10 cases (50%) achieved partial response; the ORR was 85% (17/20); one patient (5%) was stable, and two patients (10%) had disease progression (Table 2, Figure 1). The mean operation time was 276 min, and the average number of dissected lymph nodes was 19.7. The intraoperative blood loss was 184 mL, and the postoperative complication rate was 42.8% (6/14), including one case of arrhythmia, two cases of pulmonary infection, one case of hoarseness which recovered after three months, two cases of anastomotic leakage, and three cases of anastomotic stenosis (Table 3). Among them, the incidence of complications ≥ grade 3 was 20% (4/20).

**TABLE 2:** Staging and surgical information of patients who underwent surgery (*N* = 20).

NO.	Gender	Age	Tumor location	Tumor length	Clinical stages	Pathological staging	Pathological response rate	PD‐L1 CPS
1	Male	70	Middle	10	cT4N2M0, IVa	ypT0N0M0,I	pCR	5
2	Female	71	Distal	8.3	cT4N2M0, IVa	ypT0N0M0,I	pCR	30
3	Male	57	Middle	4.2	cT4N2M1a, IVb	ypT2N0M0,I	PR	3
4	Female	70	Proximal	3.0	cT4N2M0, IVa	ypT4aN1M0,IVa	SD	5
5	Female	58	Middle	14	cT4N3M0, IVa	ypT0N0M0,I	pCR	<1
6	Male	73	Middle	5.6	cT4N2M0, IVa	ypT3N1M0,IIIb	PR	15
7	Female	62	Middle	8.1	cT3N2M0, III	ypT3N0M0,II	PR	3
8	Female	67	Middle	4.9	cT3N3M0, IVa	ypT0N0M0,I	pCR	<1
9	Male	63	Middle	6	cT4N2M0,IVa	ypT0N0M0,I	pCR	15
10	Male	63	Middle	5	cT4N2M0, IVa	ypT3N0M0,II	PR	15
11	Male	69	Middle	4	cT4N0M0, IVa	ypTisN0M0,I	pCR	<1
12	Female	70	Middle	5	cT4N1M0, IVa	ypT2N1M0,IIIa	PR	<1
13	Male	74	Middle	7	cT4N2M0 IVa	ypT3N1M0,IIIb	PR	3
14	Male	74	Middle	9	cT4N1M0,IVa	ypT3N0M0,II	PR	1
15	Female	52	Middle	5.5	cT4N2M0 IVa	ypT3N0M0,II	PR	10
16	Male	66	Distal	6	cT4N0M0, IVa	ypT2N0M0,I	PR	15
17	Female	64	Middle	4	cT3N2M0, III	ypT3N2M0,IIIb	PD	12
18	Male	59	Middle	6.7	cT4N2M0 IVa	ypT3N1M0,IIIb	PD	60
19	Male	68	Middle	5.7	cT4N2M0 IVa	ypTisN0M0,I	pCR	‐
20	Male	65	Middle	8.4	cT4N2M0 IVa	ypT3N0M0,II	PR	1

Abbreviations: CPS, combined positive score; pCR, pathological complete response; PD, progressive disease; PD‐L1, programmed death‐1; PR, partial response; SD, stable disease.

**TABLE 3:** Surgical outcomes of patients who underwent surgery (*N* = 20).

Events	No. (%)
Operation time (min)	276.6 ± 63.2
Estimated blood loss in the chest (mL)	184.3 ± 76.2
Number of dissected lymph nodes	19.8 ± 8.50
Postoperative events	
Hoarseness	1 (5%)
Pneumonia	2 (10%)
Anastomotic leakage	2 (10%)
Anastomotic stenosis	3 (15%)
Arrhythmia	1 (5%)

### Safety assessment

The most common treatment‐related adverse events (TRAEs) were grade 1 or 2. The incidence of TRAE grade ≥3 was 30% (6/20), including neutropenia (5%), leukopenia (5%), and alopecia (15%) (Table 4). Among them, adverse reactions related to the immune system included reactive capillary endothelial proliferation in 14 cases (70%), decreased thyroid function in one case (5%), and acute infusion reaction to the drug camrelizumab in one case (5%). There were no surgery‐related adverse events (AEs) or treatment‐related deaths.

**TABLE 4:** Immune‐related adverse events observed after treatment with camrelizumab plus chemotherapy.

Events	Any grade	Grade 3
Reactive cutaneous capillary endothelial proliferation	14 (70%)	0
Itchy skin	1 (5%)	0
Hypothyroidism	1 (5%)	0
Acute infusion reaction	0	1 (5%)
White blood cell count decreased	9 (45%)	1 (5%)
Neutrophil count decreased	9 (45%)	1 (5%)
Thrombocytopenia	2 (10%)	0
Alopecia	9 (45%)	3 (15%)
Neurotoxic effects	7 (35%)	0
Myalgia	3 (15%)	0
Fatigue	6 (30%)	0
Increased ALT	4 (20%)	0

Abbreviation: ALT, alanine transaminase.

## DISCUSSION

**FIGURE 2:**
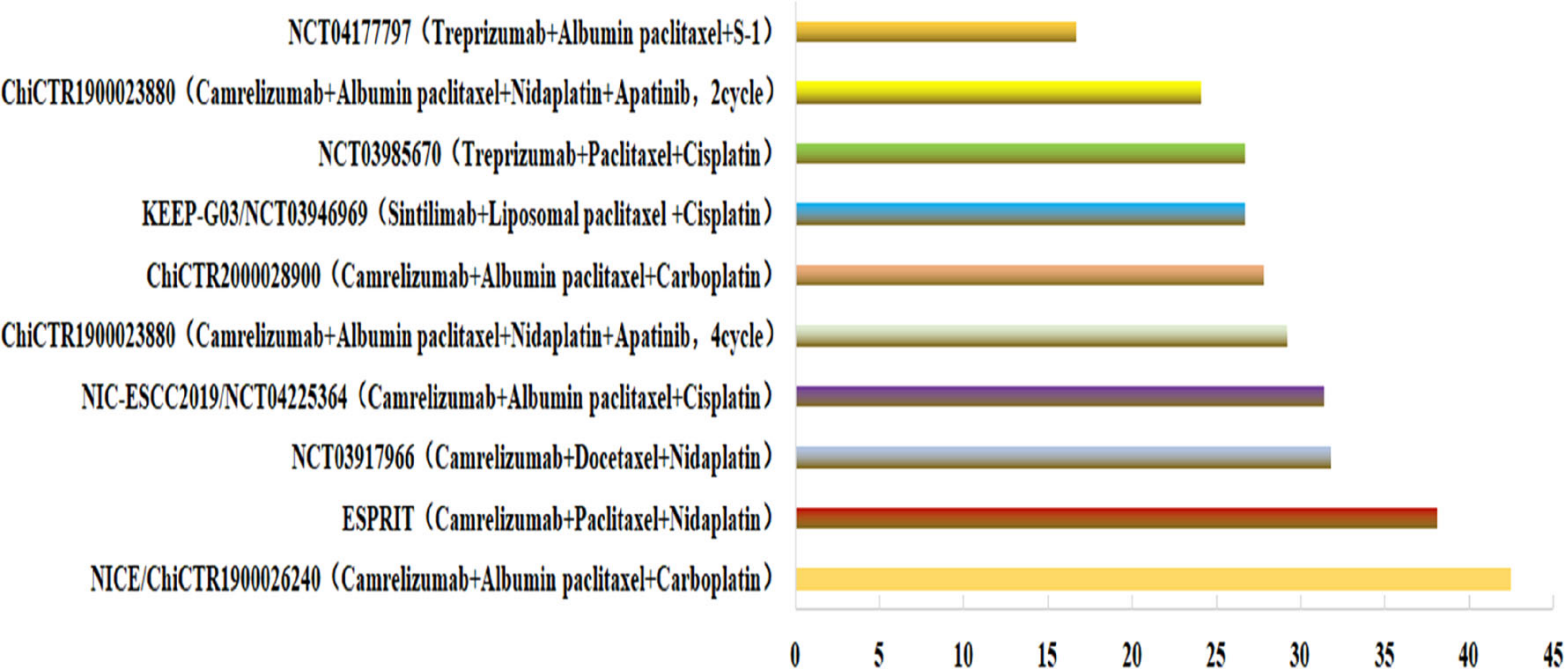
Summary chart of pathological complete response (pCR) rates in phase II clinical studies on preoperative neoadjuvant immunochemotherapy.

An ideal neoadjuvant therapy is associated with a high pCR rate, a long overall survival, low disturbance for the following operation and convenience in clinical practice. Immunotherapy has been recommended as a first‐ or second‐line treatment for patients with late‐stage or metastatic esophageal cancer. Combination therapies with immunotherapy and chemotherapy have garnered high anticipation due to their long‐term benefits and tolerability. Preclinical studies suggested that chemotherapy could stimulate the human immune system and enhance the ability of immune checkpoint inhibitors.^12^ With the continuous improvement of immunotherapy drugs, more optimal options can be provided. Currently, immunotherapy has emerged as a novel treatment modality for cancer. Various immune checkpoint inhibitors such as camrelizumab, pembrolizumab, and sintilimab have been shown to be effective and safe for the treatment of cancer.^13^, ^14^ Clinical studies such as Keynote181, Attaction3, and Escort have shown that immunotherapy significantly improves the prognosis of advanced esophageal cancer in second‐line treatment.^15^, ^16^, ^17^ Moreover, clinical studies such as Keynote590, Escort‐1st, JUPITER‐06, checkmeta648, and Orient15 have demonstrated that immunotherapy further improves both ORR and mOS, and reduces the risk of progression or death by 26%–37% in the first‐line treatment of esophageal cancer.^16^, ^17^, ^18^, ^19^, ^20^, ^21^, ^22^ Studies have indicated that taxane‐based chemotherapy induces maximum tumor cell immunogenic death and thus activates the immune microenvironment.^23^ In comparison to fluorouracil/platinum‐based combination immunotherapy, the combination of paclitaxel/cisplatin exhibits better survival benefits for patients with esophageal cancer in first‐line treatment. Increasing evidence suggests that neoadjuvant immunotherapy combined with chemotherapy can improve the prognosis of patients with locally advanced esophageal cancer.^24^, ^25^, ^26^, ^27^, ^28^, ^29^, ^30^, ^31^, ^32^, ^33^ We summarized and analyzed ongoing phase II clinical studies on neoadjuvant immunotherapy combined with chemotherapy (Table 5, Figure 2), which showed varying pCR, major pathological response (MPR), and ORR values but significant downstaging, minimal toxicity, and high R0 resection rates, as a result, more patients can achieve better results in surgery.

**TABLE 5:** Preoperative phase II clinical studies on the use of neoadjuvant immunotherapy combined with chemotherapy.

Drugs	PI	Number	Phase	*n*	Chemotherapy	Results	Publication
Camrelizumab	Wang^26^	ChiCTR1900023880	Ib	30	Albumin paclitaxel 150 mg/m^2^ Nidaplatin 50 mg/m^2^	pCR: 24.1%, MPR: 51.7% (2 cycle), pCR: 29.2%, MPR: 58.3% (4 cycle)	2021 ASCO #4047
Camrelizumab	NE^27^	NCT04225364 NIC‐ESCC2019	II	51	Albumin paclitaxel 260 mg/m^2^ Cisplatin 75 mg/m^2^	pCR: 31.4%, ORR:66.7%, MPR: 58.8%	2021 ASCO #4028
Camrelizumab	Wang^28^	NCT03917966	II	22	Docetaxel 75 mg/m^2^ Nidaplatin 75 mg/m^2^	pCR: 31.8%, R0 resection rate 100% MPR: 68.2%	2021 ASCO GI poster #222
Camrelizumab	Cheng^29^	ChiCTR2000028900	II	20	Albumin Paclitaxel 260 mg/m^2^ Carboplatin AUC = 5	pCR: 27.8%, MPR: 72.2% (+pCR) ORR:85%, DCR:100%	2021 ASCO GI Abstract #220
Camrelizumab	Li ^30^	ChiCTR1900026240 NICE	II	60	Albumin paclitaxel 100 mg/m^2^ d1,8,15, carboplatin AUC = 5	pCR: 42.5% R0 resection rate 100%	2020 ESMO Asia 2021 ASCO #4060
Camrelizumab	Ma^31^	ESPRIT	II	24	Paclitaxel 155 mg/m^2^ Nidaplatin 80 mg/m^2^	ORR:38.1%, DCR:100%, pCR: 57.1%	2021 CSCO
Sintilimab	Gu et al.^32^	CT03946969 KEEP‐G03	I/II	20	Liposomal paclitaxel 135 mg/m^2^, Cisplatin 25 mg/m^2^, S‐1 40 mg, bid, 2 cycle	pCR: 26.7%, MPR: 53.3%, R0 resection rate 100%	2020 ESMO Asia #175P
Treprizumab	Jiao et al.^34^	NCT04177797	II	24	Albumin paclitaxel, S‐1	pCR: 16.67%, MPR: 50% ORR:79.1%, DCR:100%	2020 ESMO virtual Abstract #1058P
Treprizumab	Zhao^33^	NCT03985670	II	30	Paclitaxel 150–175 mg/m^2^ Cisplatin 70–75 mg/m^2^	pCR: 26.7%, MPR: 53.3%	2021 ASCO #4051

Abbreviations: MPR, major pathological response; ORR, objective response rate; pCR, pathological complete response; PI, principle investigator.

It is noteworthy that the NICE and CHICTR2000028900 studies share the same study protocol, both employing the combination of camrelizumab, albumin paclitaxel and carboplatin. However, the pCR differed significantly (45.4% vs. 27.8%) between the two studies. The possible reason for this difference is that the NICE study included patients with a later stage (82% with T3–T4a and 100% with N2), who had a higher tumor burden, and chemotherapy promotes a greater release of tumor antigens, further enhancing the antitumor immune response. However, the NICE study found that 72.3% of patients experienced grade 3 adverse reactions, with a delay in surgery of 5.7 days, which could be attributed to variations in nutritional status in the patient group, as well as the high intensity and density of chemotherapy. Real‐world studies of patients with locally advanced resectable esophageal cancer have shown that the combination of preoperative neoadjuvant immune and chemotherapy achieves good efficacy. In a retrospective analysis by Wu et al. of 38 patients with esophageal squamous cell carcinoma treated with pembrolizumab (55.26%) and camrelizumab (31.58%), 35 (92.11%) underwent R0 resection surgery, with a pCR rate of 34.21% and an MPR rate of 42.11%. Among them, 26.32% of patients experienced postoperative complications.^35^ Shen et al. retrospectively analyzed 28 patients with locally advanced resectable esophageal cancer, and 27 patients underwent surgery, with an R0 resection rate of 96.3% (26/27), including 15 patients (55.6%) who underwent minimally invasive surgery. The postoperative pCR rate was 33.3%.^7^ To date, there have been few studies on the combination of neoadjuvant immune and chemotherapy for the conversion treatment of unresectable esophageal cancer. Fan et al. described 38 patients with locally advanced esophageal cancer (III–IVA) who underwent conversion therapy, and 27 patients underwent surgical treatment, with an R0 resection rate of 81.5%, a pCR rate of 34.21%, an MPR rate of 42.4%, and a tumor reduction rate of 65.7%.^11^

Here, we retrospectively analyzed 20 patients with locally advanced esophageal cancer who underwent preoperative chemotherapy combined with immune induction treatment and underwent surgery, including two with stage III disease, 17 with stage IVa disease, and one with stage IVb disease. The patients had poor nutritional status, and two patients presented with esophageal obstruction, for which corrective measures were given but due to toxic side effects, we reduced the dosage of carboplatin to an AUC of 3. Despite this, we achieved similar efficacy, with a pCR rate of 35%. There is controversy over the fact that two patients had postoperative pathology showing high‐grade intraepithelial neoplasia/carcinoma in situ. Because there is currently no consensus on the pathological evaluation of neoadjuvant therapy efficacy in esophageal cancer, we tentatively considered this as pCR based on the “Expert consensus on pathological evaluation of efficacy of neoadjuvant therapy for non‐small cell lung cancer”.^36^ Even if these two patients were excluded, the pCR rate of our group was still 25%.

Fan et al.^11^ reported that dense fibrous scars were found in 55.6% of the esophageal mesentery during surgery, which increased the difficulty of the surgery. We also found that 25% (5/20) of the esophageal cancer tumors were surrounded by dense fibrotic tissue, which was also observed in patients who underwent synchronous chemotherapy and radiotherapy before surgery. This is one of the reasons why some surgeons believe that preoperative synchronous chemotherapy and radiotherapy increase both the surgical difficulty and postoperative complications. Although immunotherapy mainly involves immune cell infiltration after treatment, common treatment reactions such as fibrosis, granuloma, or the presence of foam cells are also observed in the regressing tumor bed.^33^ Currently, there are no pathological data that definitively show that different treatments produce different histological changes. Therefore, we speculate that the difficulty of the surgery is not caused directly by preoperative adjuvant therapy (such as chemotherapy, radiotherapy, and immunotherapy) but is determined by the extent of tumor infiltration and treatment response at the time of diagnosis. Therefore, surgeons should adopt a positive attitude toward the use of new preoperative adjuvant therapies such as chemotherapy, radiotherapy, immunotherapy, and targeted therapy.

Although the pCR results of this study were good, the small sample size and retrospective design are likely to cause significant bias in the current results. Moreover, the lack of a control group makes it difficult to draw a definite conclusion. Therefore, more multicenter and large‐sample data are needed to support the results. We only provide a treatment idea and possibility for patients with locally advanced borderline resectable or unresectable tumors. Due to the poor overall nutritional status of these patients, it is necessary to find a balance between treatment efficacy and toxic side effects. Good conversion effects were achieved by the combination of immunotherapy drugs and reduced doses of the associated chemotherapy drugs. We believe that with further research, patients with locally advanced esophageal cancer can obtain better survival opportunities after the addition of immunotherapy to their treatment.

## AUTHOR CONTRIBUTIONS

Conception and design: Chao Li and Jianting Liu. Statistical study: Yun Chen and Yuhui Fan. Data analysis and interpretation: Shuangping Zhang and Shiping Guo. Writing – Original Draft: Shuangping Zhang and Yun Chen.

## FUNDING INFORMATION

This study was supported by Wu Jieping Medical Foundation (no: 320.6750.2021‐23‐5).

## CONFLICT OF INTEREST STATEMENT

The authors have no conflicts of interest to declare.
